# Improving healthcare delivery at a district hospital through teaching interns – A short report

**DOI:** 10.4102/phcfm.v16i1.4384

**Published:** 2024-04-26

**Authors:** Amos Mailosi, Jessie Mbamba, Carol Humphry, Anda Nindi-Nyondo, Modai C. Mnenula

**Affiliations:** 1 Department of Family Medicine, School of Medicine and Oral Health, Kamuzu University of Health Sciences, Blantyre, Malawi; 2 SEED Global Health, Malawi, South Africa

**Keywords:** clinical lecture series, clinical officer, medical assistant, family medicine

## Abstract

Every district in Malawi has at least two doctors managing the social and healthcare needs of the local population. The medical doctors at the district are involved in administrative work and have minimal time for clinical practice. As such in most district hospitals, clinical officers (COs) form the backbone of patient care provision. These are cadres that have a 3-year training in clinical medicine; they work side by side with medical assistants (MAs) and nurses. Apart from the Ministry of Health (MoH) workforce, the Department of Family Medicine (FM) of Kamuzu University of Health Sciences (KUHeS) has its main district site at Mangochi. Family physicians and residents from FM department assist in provision of mentorship and teaching to other cadres. Work-based learning requires various strategies and approaches. The experience reported here involves deliberate mentorship and support to enhance the learning of other cadres. Family medicine residents learn through the active participation in these sessions to become future consultants and leaders in primary health care.

## The context

Currently, in the Malawi healthcare system, clinical officers (COs) and medical assistants (MAs) remain the main providers of primary healthcare.^1,2^ Family medicine is relatively new to Malawi’s healthcare system.^3^ One of the aims of family medicine is to strengthen and improve the primary health care provision though collaborating, mentoring and teaching other members of the healthcare team.^4,5^ The leadership role of family physician is also one of the main competencies. While this report focuses on teaching and mentoring roles, it should be noted that these roles are made possible and anchored by the leadership role.^6^

Every district in Malawi has at least two doctors managing the social and healthcare needs of the local population. The medical doctors in the district are involved in administrative work and have minimal time for clinical practice. As such, in most district hospitals COs form the backbone of patient care provision. These are cadres that have a 3-year training in clinical medicine; they work side by side with MAs and nurses.^2^ The MAs have 2-year training in clinical medicine and are awarded with a certificate in clinical medicine after graduating.

Clinical officers learn the science of medicine and put it into clinical practice in much the same way doctors do in medical schools although CO study involves less detail because of the shorter training schedule. They are awarded a diploma in clinical medicine when they graduate. After graduating, they must complete a year of clinical practice in a district hospital under the supervision of qualified COs or available medical doctors; this is called a CO internship. Medical Assistants work under the supervision of COs in managing non-complicated medical cases. They may run rural primary care clinics, with or without a CO on site, and they refer complicated conditions to the district hospitals. Intern medical assistants (IMOs), like intern clinical officers (ICOs), do their internship at the district hospitals.

Apart from MoH workforce, the Department of Family Medicine (FM) of Kamuzu University of Health Sciences (KUHeS) has its district base at Mangochi District Hospital (MDH). Family physicians and residents from FM department assist in provision of mentorship and teaching to other cadres. One must understand that work-based learning takes various strategies and approaches.^7^ Mangochi District Hospital interns (ICOs or IMAs) are the front-liners for the 24 h on-call team. However, observations from patient handover meetings between on-call and day shift team revealed a gap in medical knowledge among interns. Thus, the clinical lecture series (CLS) specifically focused on management of common primary care conditions. Clinical lecture series are sessions organised by the family medicine department that are done every 2 weeks with the aim of improving the medical knowledge and skills of the IMOs and ICOs. This report focuses on deliberate mentorship and support to address the gaps identified in the medical knowledge and skills of IMOs and ICOs. Family medicine residents also strengthen their teaching skills through facilitation of CLS session as future consultants and leaders.

In brief, the following gaps were identified before starting CLS sessions: (1) inadequate knowledge in making diagnosis and management of the common conditions presenting to MDH, (2) lack of practical skills in the common procedures, such as thoracentesis and others and (3) lack of use of evidence-based medicine and its application to the local clinical setting.

## The intervention

In 2016, the FM department joined with MoH, taking an active role in the training of the intern COs and intern MAs through CLS. Later, interns from laboratory and pharmacy also joined the classes. To make the teaching more appropriate and useful, the interns themselves choose the topics to be covered and who among them will prepare the presentation. The job of a family physician or resident is to facilitate the class, adding more knowledge to the interns’ presentations and making corrections. To avoid disrupting daily work schedules, these classes take place during lunch hour from noon to 13:30. In the beginning, the turnout was not very good, with perhaps half of the interns attending, that is, less than 15. At very period, MDH tends to have about 30 interns. However, these lectures have grown into one of the most attended classes. Currently, more than 90% of the interns at MDH attend, that is, 28–30 interns.

The teaching focuses on basic medical science, evidence-based medicine, best clinical practice as well as clinical skills; common primary health care patient conditions are included. The practical sessions include common emergency room interventions such as cardiopulmonary resuscitation (CPR), advanced cardiac life support (ACLS), common procedures such as thoracentesis and suturing techniques. Manikins are used in some of these sessions. The sessions are relevant to the interns’ clinical practice. The knowledge gained from these lectures includes how to apply the skills to the clinical scenarios that interns come across quite often in their hospital work. Practical sessions, special physical examination or practical sessions are included. Scientific evidence and management of diseases are adapted to the resource-limited setting of the district hospital.

## The effect

Through interviews with Intern COs (ICOs) and Intern MAs (IMAs) who have been part of this CLS, it was revealed that the teaching sessions had been very important in their professional development and healthcare provision. They liked the fact that they were preparing the topics and presentations. The classes take a form of a flipped class, in which learners are given material and prepare before the class is delivered; the active involvement in their own teaching was the most cited positive thing in the feedback interviews. Some other important points were change of clinical practice: on learning new information such as the management of a particular condition, participants reported that they changed their clinical practice according to the newly gained knowledge. They reported:

- improved confidence in their ability to effectively manage difficult cases.
- improved teamwork and collaboration among the interns.

We conducted two focus group discussions (FGDs). One group consisted of 15 senior interns or qualified COs or MAs, the other one consisted of 15 junior internees at MDH who were regular attendees of CLS. Both groups expressed that CLS was important to their career development. They overall agreed it was one feature that attracted them to come learn at MDH and it improved their ability to care for patients. Table 1 summaries themes and comments on outcomes unveiled from FGDS on CLS sessions.

**TABLE 1:** Themes reported by interns on clinical lecture series conducted at Mangochi District Hospital.

Theme	Outcome
Value of flipped classroom approach	Most interns highlighted that they learnt a lot as they prepared the presentations prior to session time
Perceived improved clinical practice	Their confidence and knowledge in managing difficult patients improved
Improved teamwork and collaboration between Family medicine consultants, registrars and interns	Interns reported prior interactions during CLS sessions made it easy for them to consult FM consultant or registrars when stuck during patient management in the wards
Influence on career development	Influenced the decision to choose MDH for their internship

MDH, Mangochi District Hospital; CLS, clinical lecture series; FM, family medicine.

Here are couple samples of the extracts from the FGDs showing influence on career development and perceived improved clinical practice:

‘I decided to do my internship at MDH after my friends had told me it was a good place for learning; I was told, with the presence of Family Medicine doctors, there is a very good intern-mentorship program.’ (male, junior intern clinical officer, 26 years old)

‘CLS has assisted me to always look beyond the guidelines and find scientifically sound alternatives to guidelines when I am stuck.’ (male, clinical officer, 29 years old)

Over the years the CLS have become one of the unique features of MDH in terms of interns’ mentorship.

## Strengths and weaknesses of the method

The feedback shows that CLS capacitated lower cadres (ICOs and IMAs) of Mangochi District Hospital in healthcare provision. Clinical lecture series have proved as effective learning tool during internship training. The classes have also improved the relationship between the MoH team and the FM department members. The improvement in patient management cannot be over-emphasised. We have seen a general change in the perception by the MoH team of our FM presence at MDH; while in the beginning, we could be perceived as a parallel structure, now the CLS is recognised as part of the formal teaching programme for internees at MDH. The CLS are very important especially to the residents of FM; their active participation and facilitation of the CLS prepare them for the lifetime roles of a teacher, a consultant and a leader in primary health care.

Following internship, the relation continues for those clinicians who remain to work at MDH. Our ability to provide meals during the sessions is supported by the NGO Seed Global Health, which has sustained the presentation of these lectures for years. However, this may not be feasible in settings with financial constraints and without external support. So, a different arrangement or time may be needed.

## Conclusion

The CLS programme has proved to be a good tool to facilitate mentorship of junior cadres in the primary health care at district hospital. The presence of family medicine at Mangochi District Hospital has contributed to teaching and improved skills of the COs and MA internees.

The authors would like to acknowledge the contribution of SEEDS Global Health for funding the continuous lecture series (CLS), through the Department of medicine; we also acknowledge the contribution by the Department of Family Medicine in delivering the lectures; we acknowledge all current and previous interns at Mangichi District Hospital for their very important feedback on CLS.
